# Free heme exacerbates colonic injury induced by anti-cancer therapy

**DOI:** 10.3389/fimmu.2023.1184105

**Published:** 2023-06-05

**Authors:** Philippa Seika, Monika Janikova, Sahana Asokan, Lubica Janovicova, Eva Csizmadia, Mckenzie O’Connell, Simon C. Robson, Jonathan Glickman, Barbara Wegiel

**Affiliations:** ^1^ Department of Surgery, Division of Surgical Sciences, Cancer Research Institute, Beth Israel Deaconess Medical Center, Harvard Medical School, Boston, MA, United States; ^2^ Institute of Molecular Biomedicine, Faculty of Medicine, Comenius University, Bratislava, Slovakia; ^3^ Division of Microbiome and Cancer, German Cancer Research Center (DKFZ), Heidelberg, Germany; ^4^ Faculty of Biosciences, Heidelberg University, Heidelberg, Germany; ^5^ Department of Anesthesia, Beth Israel Deaconess Medical Center, Harvard Medical School, Boston, MA, United States; ^6^ Department of Pathology, Beth Israel Deaconess Medical Center, Harvard Medical School, Boston, MA, United States

**Keywords:** gastrointestinal syndrome, radiation enteritis, free heme, hemopexin, heme oxygenase-1

## Abstract

Gastrointestinal inflammation and bleeding are commonly induced by cancer radiotherapy and chemotherapy but mechanisms are unclear. We demonstrated an increased number of infiltrating heme oxygenase-1 positive (HO-1+) macrophages (Mø, CD68+) and the levels of hemopexin (Hx) in human colonic biopsies from patients treated with radiation or chemoradiation versus non-irradiated controls or in the ischemic intestine compared to matched normal tissues. The presence of rectal bleeding in these patients was also correlated with higher HO-1+ cell infiltration. To functionally assess the role of free heme released in the gut, we employed myeloid-specific HO-1 knockout (*LysM-Cre : Hmox1^flfl^*), hemopexin knockout (*Hx^-/-^*) and control mice. Using *LysM-Cre : Hmox1^flfl^* conditional knockout (KO) mice, we showed that a deficiency of HO-1 in myeloid cells led to high levels of DNA damage and proliferation in colonic epithelial cells in response to phenylhydrazine (PHZ)-induced hemolysis. We found higher levels of free heme in plasma, epithelial DNA damage, inflammation, and low epithelial cell proliferation in *Hx^-/-^* mice after PHZ treatment compared to wild-type mice. Colonic damage was partially attenuated by recombinant Hx administration. Deficiency in *Hx* or *Hmox1* did not alter the response to doxorubicin. Interestingly, the lack of Hx augmented abdominal radiation-mediated hemolysis and DNA damage in the colon. Mechanistically, we found an altered growth of human colonic epithelial cells (HCoEpiC) treated with heme, corresponding to an increase in *Hmox1* mRNA levels and heme:G-quadruplex complexes-regulated genes such as *c-MYC, CCNF*, and *HDAC6.* Heme-treated HCoEpiC cells exhibited growth advantage in the absence or presence of doxorubicin, in contrast to poor survival of heme-stimulated RAW247.6 Mø. In summary, our data indicate that accumulation of heme in the colon following hemolysis and/or exposure to genotoxic stress amplifies DNA damage, abnormal proliferation of epithelial cells, and inflammation as a potential etiology for gastrointestinal syndrome (GIS).

## 1 Introduction

Chemoradiation (CRT) remains the standard recommended treatment modality for oncological diseases and can induce durable responses in a wide range of human cancers. However, CRT is commonly associated with side effects that limit therapy completion. Nearly 30% of cancer patients treated with chemotherapy develop severe treatment-limiting toxicities (1, 2). The more frequent gastrointestinal toxicities of chemotherapy include nausea, vomiting, diarrhea, and infections, especially in the setting of neutropenia. Chemotherapeutic agents, including doxorubicin, have been implicated in three patterns of colitis: pseudomembranous colitis, neutropenic enterocolitis, and ischemic colitis (3, 4). Similarly, acute effects of radiation exposure to the colon can include nausea, vomiting, diarrhea, bleeding, abdominal pain, and dehydration, which constitute symptoms of an iatrogenic condition known as gastrointestinal syndrome (GIS) (5). Severe radiation exposure can cause more serious complications, such as damage to the intestinal lining, leading to the release of toxins into the bloodstream and potentially life-threatening conditions (6).

The cellular and molecular mechanisms of these adverse events are not well understood, precluding their optimal clinical management (6, 7). Chemotherapy (*i.e.*, doxorubicin) or radiation can damage and eradicate not only cancer cells but also normal cells such as red blood cells (RBC) or epithelial cells, causing the release of danger-associated molecular patterns (DAMPs). Among DAMPs, free heme can amplify DNA damage and inflammation induced by chemotherapy drugs (8). This is thought to occur because free heme can generate reactive oxygen species (ROS), which can cause oxidative damage to DNA and other cellular components and activate immune cells, leading to inflammation. We have previously demonstrated that enzymatically active HO-1, protects normal cells from doxorubicin-induced cell death (9). In contrast, HO-1 by generating carbon monoxide amplified cancer cell death in response to anti-cancer therapy (10). Our recent studies have also demonstrated that exogenous heme induces DNA damage in various cell types *in vitro* and *in vivo* (9, 11, 12). Further, heme degradation by the activity of HO-1 prevents cellular senescence in Mø to suppress chronic DNA damage and proliferative responses in the gut in HO-1-deficient mice (9).

Heme is a high-energy prosthetic group of hemoproteins with varied functions ranging from gas carriers (*i.e*., hemoglobin), transcription factors (*i.e*., neuronal PAS domain protein 2), and cytochromes to redox enzymes (9). As a pro-inflammatory molecule, heme plays a central role in gut homeostasis and enteric inflammation. Free heme is scavenged by hemopexin (Hx) and its receptor on myeloid cells, CD91/LRP. Subsequently, free heme is degraded by the activity of heme oxygenases (HO-1 and HO-2) generating carbon monoxide, biliverdin, and iron (13, 14). Recent research has highlighted the significance of heme metabolism in colonic epithelial cells (9). Excessively high levels of heme and iron during anti-tumor treatment, and attenuated levels of HO-1, may lead to oxidative modifications of lipids and proteins as well as DNA damage, resulting in inflammation and accelerated organ injury (15). Recent studies also emphasize the role of HO-1-expressing CX3CR1+ intestinal Mø in resolving gut inflammation and colitis as well as in the regulation of polyps and cancer growth in the models of colon carcinogenesis (16).

Heme is a well-established pro-inflammatory signaling molecule (9, 11, 12). DAMPs such as heme are generated during hemolysis and have been shown to trigger inflammation in the gastrointestinal tract through the Absent in Melanoma 2 (AIM2) and Toll-Like Receptor 3 or 4 signaling pathways (17–19). In addition, heme is a significant promoter of nicotinamide adenine dinucleotide phosphate oxidase-dependent ROS formation in intestinal cells (20). Together with ROS, heme promotes colonic cell permeability, migration, and proliferation, as well as interleukin 6 production (21). Here, we have examined how free heme, which is generated in the gut after bleeding or cell death following colonic injury, exacerbates damage and inflammation in the colon basally and in response to anti-cancer treatment. High levels of free heme in response to bleeding, hemolysis, trauma, and local or systemic cell damage (as in the case of radiation, chemotherapy, or infection) could overwhelm the capacity of HO-1 and Hx. The mechanistic function of free heme released in the colon as a consequence of anti-cancer therapy remains largely unknown. Studies have illustrated the protective role of HO-1 against ischemia reperfusion-mediated intestinal injury and in colitis and maintenance of gut homeostasis by regulating macrophage phagocytosis (22, 23). In response to damage, functionally impaired immune cells, including Mø, infiltrate the colonic epithelium.

In this study, we determined the tissue expression of HO-1 and DNA damage marker (γH2AX) in colorectal biopsies from patients with GIS. We showed the importance of intestinal bleeding and HO-1 induction in the colons of patients treated with anti-cancer therapy. We further defined the role of myeloid-derived HO-1 and Hx in mouse models of hemolysis, radiation, and chemotherapy. We demonstrated that deletion of HO-1 in myeloid cells or lack of Hx in the model of hemolysis leads to amplification of DNA damage and proliferation, thus, altering the homeostasis in the colon. Despite increased free heme in the gut in response to doxorubicin treatment, we have not found a major role of *Hmox1* in myeloid cells in this model, in agreement with data obtained in Hx-deficient mice. However, we showed the importance of Hx in the gut in response to radiation-induced hemolysis and injury. Our study provides a novel explanation of how alterations in the free heme levels in the gut (due to lack of Hx or HO-1) may impact colonic injury and the process of healing in response to anti-cancer therapy.

## 2 Results

### 2.1 Increased number of HO-1+ Mø in human colon biopsies obtained from patients treated with radiotherapy, chemotherapy, or diagnosed with colon ischemia with active rectal bleeding

**Figure 1:**
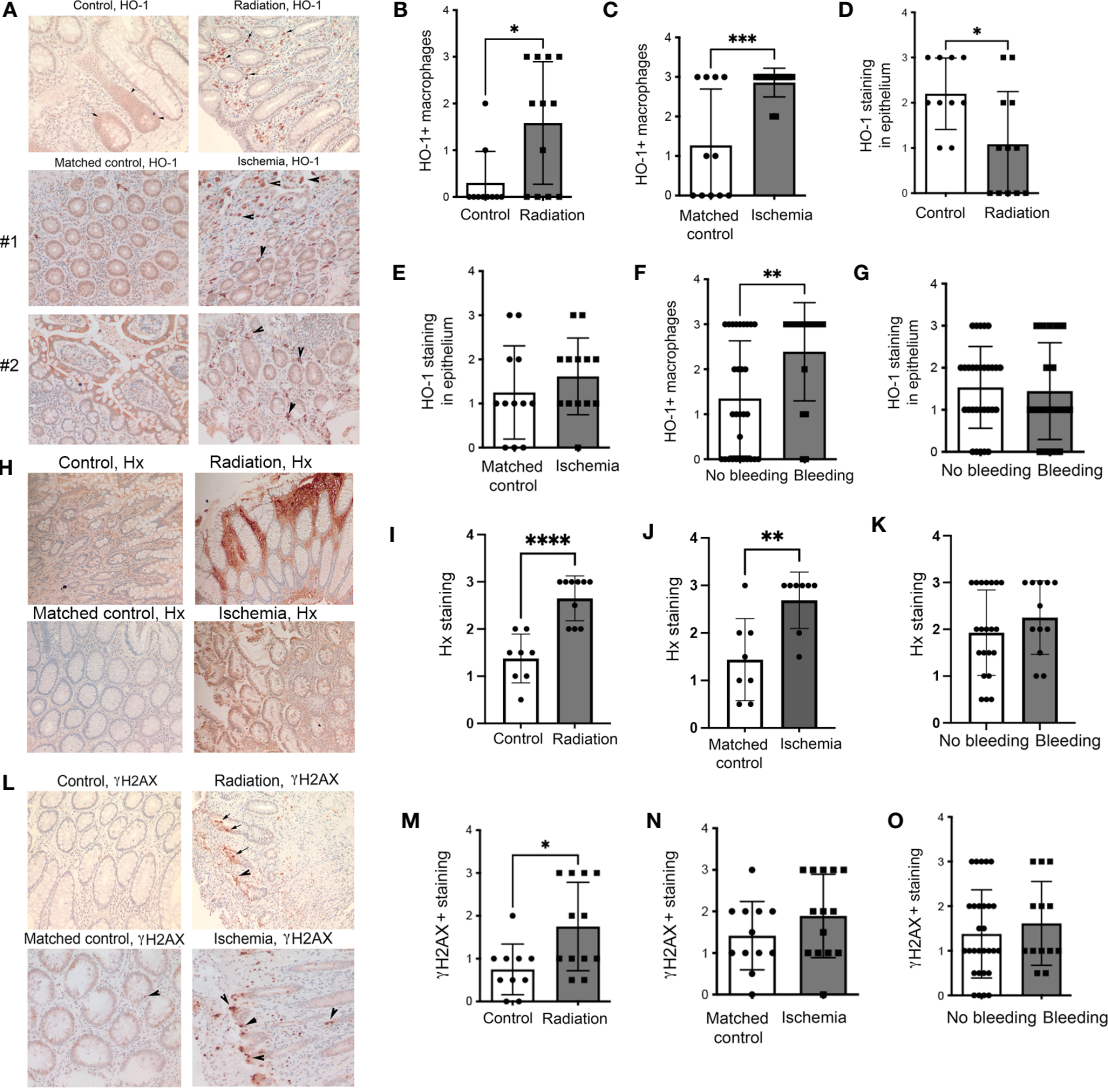
Expression of HO-1, Hx, and γH2AX in human intestinal biopsies from patients treated with radiation or suffering from intestinal ischemia. **(A)** Representative immunostainings of HO-1 in colonic biopsies from cancer patients treated with radiotherapy (“Radiation”) or diagnosed with colonic ischemia (ischemic and matched non-ischemic control tissue regions are shown). Arrows indicate HO-1+ Mø. Staining from two patients with ischemic colons and matched normal mucosa are shown. **(B–E)** Analyses of the HO-1 staining intensities in the following groups: n=11 control (untreated), n=12 irradiated, and n=11 with intestinal ischemia. Mean values +/- SD are shown. *p<0.05, **p<0.01, ***p<0.001. **(F, G)** Analyses of samples based on the presence of colonic bleeding. **(H–J)** Immunostaining of colonic biopsies as in **(A)** with antibodies against Hx. The representative staining is shown in **(H)** Analysis of n=11-12 cases is shown in **(I, J)**. ****p<0.001, **p<0.01. Mean values +/- SD are shown. Analyses of samples based on the presence of colonic bleeding is shown in **(K)**. **(L–N)** Immunostaining of colonic biopsies as in **(A)** with antibodies against DNA damage marker γH2AX. The representative staining is shown in **(L)**. Analysis of n=11-12 cases is shown in **(M, N**) *p<0.05. Mean values +/- SD are shown. Analyses of samples based on the presence of colonic bleeding is shown in **(O)**.

We first analyzed human colon biopsies obtained from cancer patients who underwent treatment with radiotherapy (n=12), untreated colorectal cancer patients (n=11), and those diagnosed with colon ischemia (n=11). Colon ischemia was diagnosed in postoperative histopathology in all cases in the last group. The colonic tissues were stained for HO-1 and γH2AX. We found high levels of HO-1+ Mø in the irradiated colons and ischemic regions of the colon but not in matched non-ischemic tissues from the same patient (**Figures 1A–C**). In ischemic regions, majority of CD68+ Mø expressed HO-1 unlike in matched non-ischemic colons (**Supplementary Figure 1**). Interestingly, the levels of HO-1 in the epithelium were lower in the irradiated colon and slightly increased in the ischemic regions of epithelium compared to matched control tissue (**Figures 1D, E**). Further, nine patients in the irradiated and ischemic groups exhibited rectal bleeding, defined clinically as presenting with either macroscopic hematochezia or fecal occult blood positivity. This cohort showed higher infiltration of HO-1+ Mø but no change in the epithelial HO-1 levels compared to those without bleeding (**Figures 1F, G**). Since heme is scavenged by Hx, we stained the same tissue with an antibody against Hx (**Figure 1H**). We showed a significant increase of Hx staining in colons from cancer patients who underwent treatment with radiotherapy (**Figures 1H, I**) as well as in the areas of the ischemic colon compared to matched non-ischemic tissues from the same patient (**Figures 1H, J**). No difference in the Hx staining was seen in the cohorts with or without bleeding (**Figure 1K**).

As expected, radiation significantly increased DNA damage (γH2AX) in the colons (**Figures 1L, M**). A non-significant trend towards higher γH2AX staining was observed in ischemic colons compared to matched non-ischemic tissue (**Figures 1L, N**). There was no difference in γH2AX staining between the colons from patients with or without rectal bleeding (**Figure 1O**). These data suggest the importance of HO-1+ Mø and Hx upon the genotoxic or ischemic stress and a possible association of HO-1 expression with bleeding (indicating a presence of free heme) in the colon.

**Figure 2:**
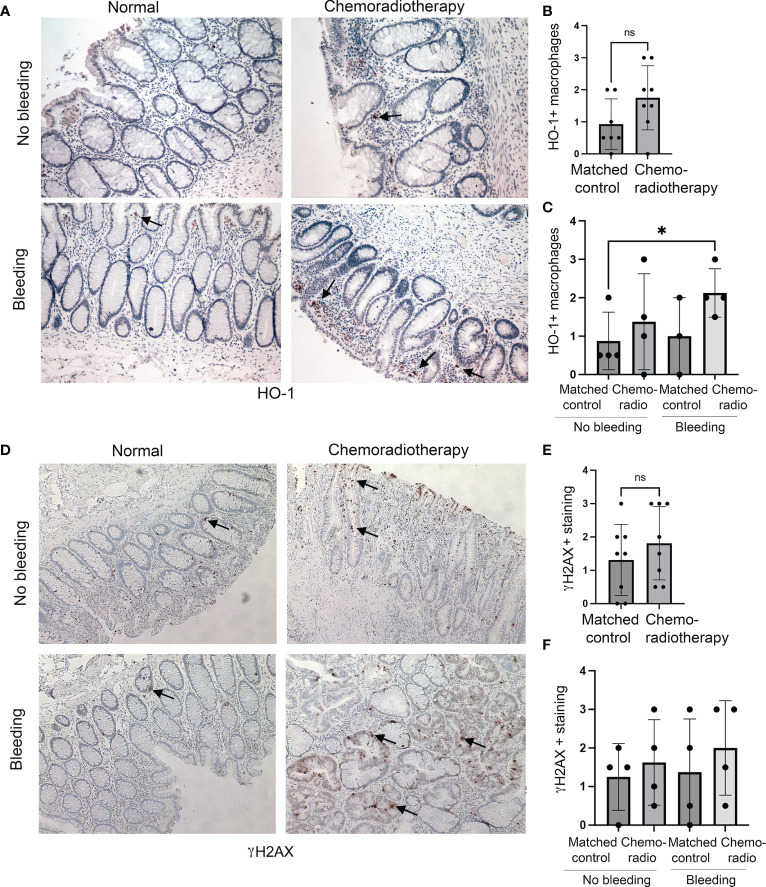
Expression of HO-1 and γH2AX in human colonic biopsies from patients treated with CRT. **(A–C)** HO-1 immunostainings of CRT treated and normal mucosa from n=8 patients with rectal tumors. Representative staining is shown in **(A)**. Evaluation of HO-1 staining in Mø is shown in **(B)** and qualitative evaluation of the staining based on the rectal bleeding status is shown in **(C)**. Mean values +/- SD are shown. *p<0.05. ns= not statistically significant. **(D–F)** Immunostainings of γH2AX of chemotherapy and normal mucosa from n=8 patients with rectal tumors treated with radiation therapy. Representative staining is shown in **(D)**. Evaluation of γH2AX staining is shown in **(E)** and statistical analysis of the staining based on clinically diagnosed rectal bleeding is shown in **(F)**. Ns, not significant.

We have also investigated colon tissues from rectal cancer patients (n=8) treated with CRT. CRT regimen was determined individually by institutional tumor board recommendation and consisted of radiation with up to 50.4Gy in conjunction with either 5-FU or capecitabine. We stained the sections of the matched injured (after CRT) and uninjured (before CRT) tissues from the same patients with antibodies against HO-1 and γH2AX (**Figure 2**). We found a trend of an increased number of HO-1+ Mø infiltrating the colon upon CRT compared to the matched control samples (**Figures 2A, B**) in both patients either with or without rectal bleeding (**Figure 2C**). Importantly, the highest infiltration of HO-1+ Mø was detected in the colons of patients treated with CRT and who also presented with rectal bleeding (**Figure 2C**). A slight increase was seen in patients treated with chemoradiation and without rectal bleeding (**Figure 2C**). This suggests that the effect of CRT on HO-1+ Mø infiltration might be dependent of rectal bleeding. There was no difference in epithelial HO-1 expression between the groups (data not shown). Further, levels of γH2AX varied in the colons of treated patients (**Figures 2D, E**) and did not correlate with the incidence of rectal bleeding (**Figure 2F**). These data support the role of HO-1+ Mø infiltration both in response to radiation as well as chemoradiation (**Figures 1**, **2**).

### 2.2 Increased DNA damage and proliferation in the colons of myeloid-specific Hmox1-knockout mice in models of heme-associated injury

**Figure 3:**
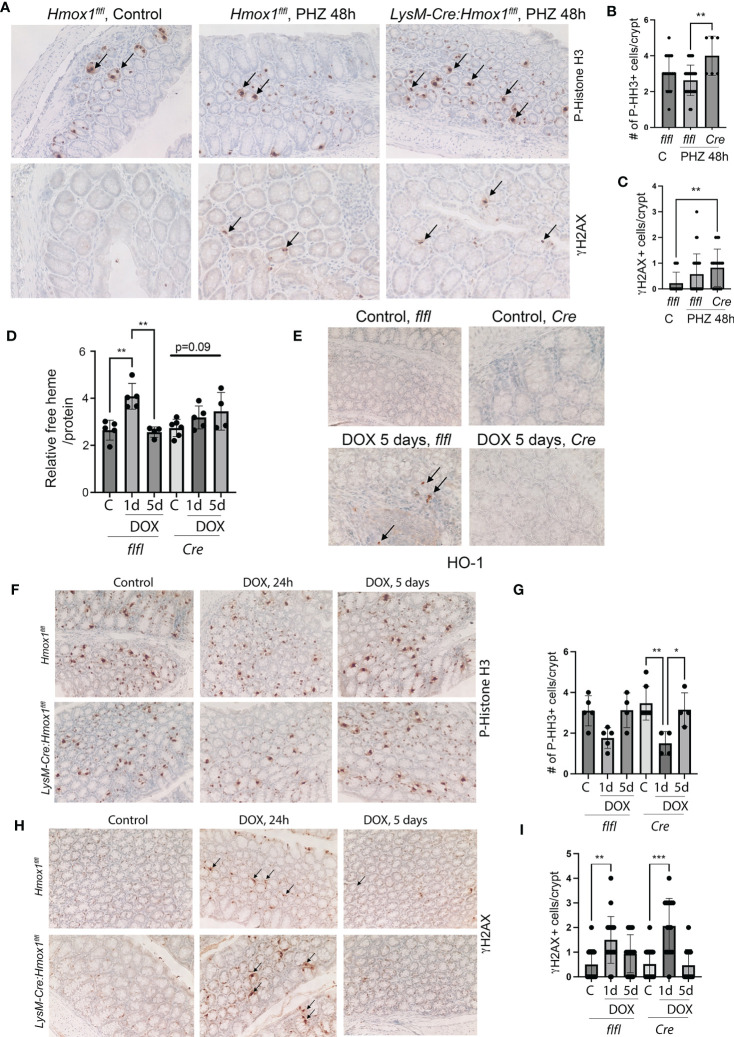
The role of Mø-expressed HO-1 in modulating DNA damage and proliferation of colonic epithelial cells in *in vivo* model of hemolysis and doxorubicin. **(A–C)** Immunohistochemical analyses of P-HH3 (a marker of proliferation) **(A, B)** and γH2AX (a marker of DNA damage) **(A, C)** in colon samples isolated from *LysM-Cre : Hmox1^flfl^* and *Hmox1^flfl^* mice treated with PHZ (150 mg/kg, i.p.) and harvested at 48 hours after treatment. A p-value of <0.05 was considered significant. *p<0.05, **p<0.01. n=4-7 mice per group. Multiple FOVs were evaluated for quantification of the number of positive cells per crypt. **(D)** Heme levels were measured in the colon lysates from mice treated as above. Relative heme levels (μmol) per protein concentration are shown **p<0.01. **(E)** Immunohistochemical analysis of HO-1 expression in the colons of mice treated as in **(D)** Representative pictures are shown. **(F–I)** Immunohistochemical analyses of P-HH3 (a marker of proliferation) **(F, G)** and γH2AX (a marker of DNA damage) **(H, I)** in colon samples as in **(D)** Arrows indicate positive staining. The quantification and statistical analysis are shown in G and **(H)** Mean values +/- SD are shown. *p<0.05, **p <0.01, ***p<0.0001.

Since the number of HO-1+ Mø was strongly increased in the colons of patients with rectal bleeding after anti-cancer therapy, we employed two models of colonic injury in mice lacking HO-1 in myeloid cells (*LysM-Cre : Hmox1^flfl^*; *Cre*) and control mice (*Hmox1^flfl^*). In the first model, we used PHZ to induce systemic hemolysis and colonic damage (**Figures 3A–C**). In the second model, we used a single dose of doxorubicin to promote DNA damage, cell death, and release of heme in the colon (**Figures 3D–I**). We found elevated DNA damage (γH2AX, **Figures 3A, C**) and proliferation of epithelial cells (P-Histone H3, **Figures 3A, B**) in mice lacking *Hmox1* in myeloid cells (*LysM-Cre : Hmox1^flfl^*; *Cre*) compared to control *Hmox1^flfl^* mice treated with PHZ (**Figures 3A–C**). These data indicate the importance of clearance of heme by HO-1 in myeloid cells, which may otherwise cause abnormal DNA damage and proliferation responses in the colon. To further investigate the role of HO-1 in myeloid cells, we treated *LysM-Cre : Hmox1^flfl^* and *Hmox1^flfl^* mice with 8 mg/kg doxorubicin (1x, *i.v*.) (n=4-6 mice/group, including females and males) and harvested colons at days 1 and 5 after treatment (**Figures 3D–I**). No differences were seen between body weights or white blood cells (WBC) levels between the genotypes or treatments (**Supplementary Figures 2A, B**). Interestingly, the lack of myeloid-derived HO-1 resulted in increased platelet (PLT), hemoglobin (Hb), and hematocrit (HCT) levels in untreated mice (**Supplementary Figures 2C–E**). Doxorubicin treatment led to a significant increase in free heme in colons with a peak at 1 day and returned to baseline at day 5 in *Hmox1^flfl^* mice (**Figure 3D**). The levels did not return to basline in *LysM-Cre : Hmox1^flfl^* mice at day 5 after treatment with doxorubicin (**Figure 3D**). The number of infiltrating HO-1+ cells was increased in the colon of *Hmox1^flfl^* control mice at 5 days after treatment with doxorubicin (**Figure 3E**). We found significant decrease in proliferation in the colons of *LysM-Cre : Hmox1^flfl^* in response to doxorubicin at day 1, which returned to a baseline level at day 5 (**Figures 3F, G**). However, no difference in proliferation was seen between the genotypes (**Figure 3F, G**). Doxorubicin treatment led to an increase in DNA damage at day 1 as measured by γH2AX staining in both groups (**Figures 3H, I**), but no difference was seen between the genotypes. These data suggest that the presence of HO-1+ infiltrating cells and free heme in the colon in the GIS model might be important for colonic homeostasis and healing in response to hemolysis/bleeding but not in response to doxorubicin-mediated injury.

### 2.3 Increased hemolysis in Hx KO mice is associated with the induction of DNA damage and poor proliferation of colonic epithelial cells

**Figure 4:**
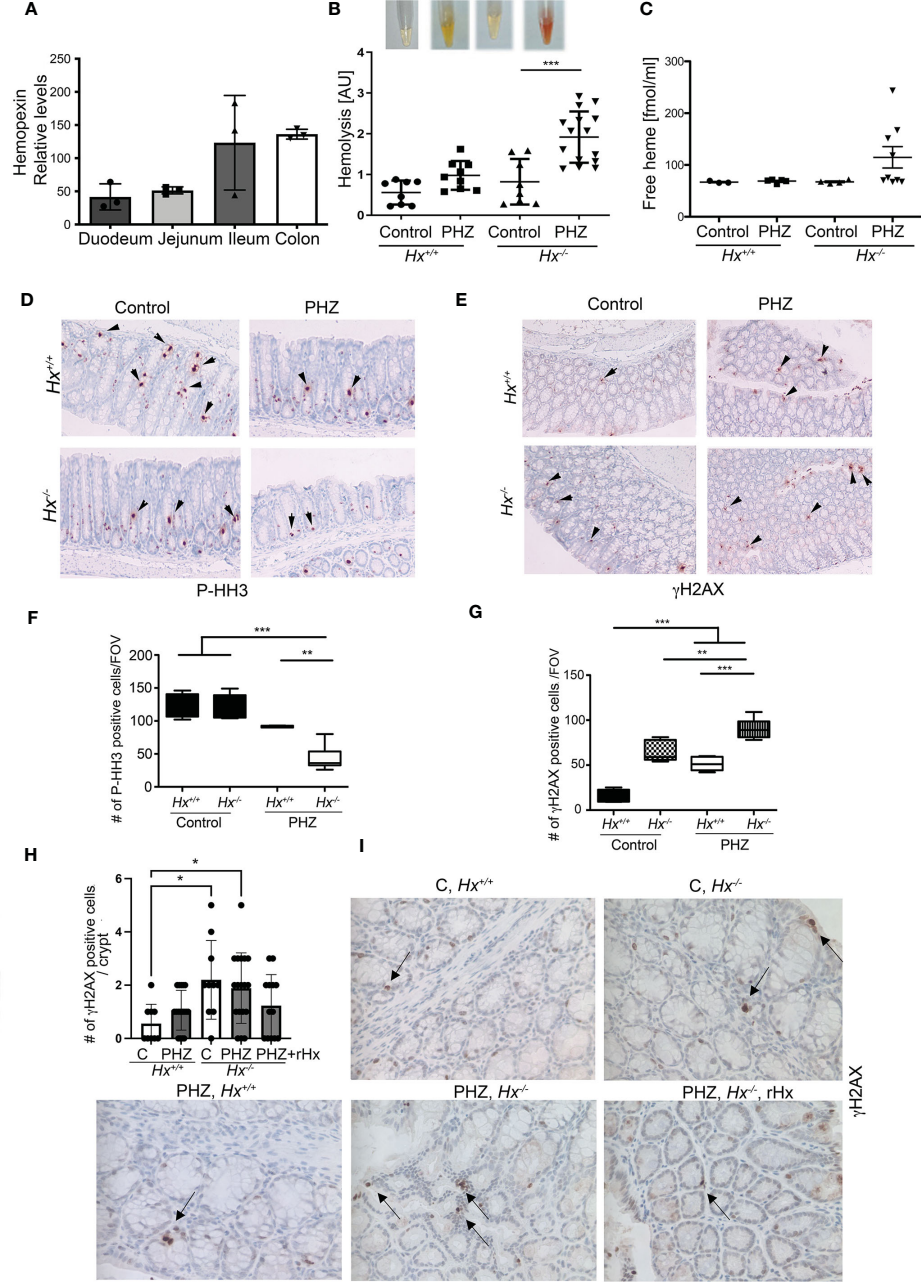
Lack of Hx leads to colonic inflammation and DNA damage *in vivo*. **(A)** *Geo* profiles of relative expression of Hx in the mouse intestinal tract based on the Affymetrix MuU74v2 Gene chip set (24). N=3 per group. **(B, C)** *Hx^-/-^* and *Hx^+/+^* mice were treated with PHZ (100 mg/kg, *i.p*.) for 48 h. Hemolysis was measured in the plasma by absorbance at 420 nm, detecting primarily free heme **(B)**. Mean values +/- SD are shown. n=3-7 mice per group. *p<0.05, **p<0.001. Free heme was measured in the same samples by BioVision kit and the data are shown in **(C)**. n=7-13 mice per group. **(D–G)** Immunohistochemical staining of P-HH3 (a marker of proliferation) **(D)** and γH2AX (a marker of DNA damage) **(E)** of the colonic tissues from *Hx^-/-^* and WT mice treated with PHZ (100 mg/kg, *i.p*.) and harvested at day 5. 200x magnification. **(F, G)** The quantifications of the number of cells positive for P-HH3 **(F)** or γH2AX **(G)** in the colons of mice treated as described in **(B)** n=3 male mice per group; 2-3 sections were analyzed in **(B)** FOV-field of view. Mean values +/- SD are shown. **p<0.01; ***p<0.001. **(H, I)** Immunohistochemical staining of γH2AX (a marker of DNA damage) of the colonic tissues from *Hx^-/-^* and WT mice treated with PHZ (100 mg/kg, *i.p*.) ± recombinant Hx (rHx, 2.5 mg/kg, i.v.) and harvested at day 2. Mean values +/- SD are shown. N=3-8 mice per group. *p<0.05.

By analyzing *Geo* profiles, we found high levels of *Hx* mRNA in the mouse ileum and colon compared to other parts of the intestine (**Figure 4A**). Previous studies indicated that *Hx ^-/-^* mice had more severe hemolysis in response to hemolysis induced by PHZ injection than wild type mice (12, 25). To define the role of Hx in GIS models and the connection between increased levels of free heme and colonic damage, we used *Hx ^-/-^* mice treated with PHZ (100 mg/kg*, i.p*.) (**Figure 4**). Control and PHZ-treated *Hx ^-/-^* mice had significantly smaller spleens compared to wild type mice (**Supplementary Figure 3A**). The levels of HO-1 and hemoglobin (Hb) were highly elevated in *Hx^-/-^* mice (**Supplementary Figure 3B**) due to elevated hemolysis and free heme levels in response to PHZ treatment (**Figures 4B, C**). Further, free heme promoted DNA damage in the spleen (**Supplementary Figures 3C, D**), similar to our previous report in cancer model (11). We observed a slight decrease in cell proliferation in the colons of *Hx^-/-^* mice treated with PHZ compared to wild-type (WT) mice (**Figures 4D, F**). The decrease in P-Histone H3 (P-HH3) staining corresponded to an increase in γH2AX staining in colons of *Hx^-/-^* mice treated with PHZ (**Figures 4D–G**). We found a significantly higher number of γH2AX+ cells in the colon of *Hx^-/-^* mice basally and after treatment with PHZ (**Figures 4E, G**). We also investigated the efficiency of recombinant Hx (rHx) to revert colonic injury following PHZ treatment in *Hx^-/-^* mice (**Figures 4H, I**). One dose of rHx (2.5 mg/kg, *i.v*.) did not significantly suppress the DNA damage as assessed by the level of γH2AX staining in *Hx^-/-^* mice treated with PHZ compared to untreated mice (**Figures 4H, I**). These data support a significant role of Hx-mediated protection in the colon in response to hemolysis and elevated free heme levels.

### 2.4 Hx deletion does not impact doxorubicin-induced DNA damage

**Figure 5:**
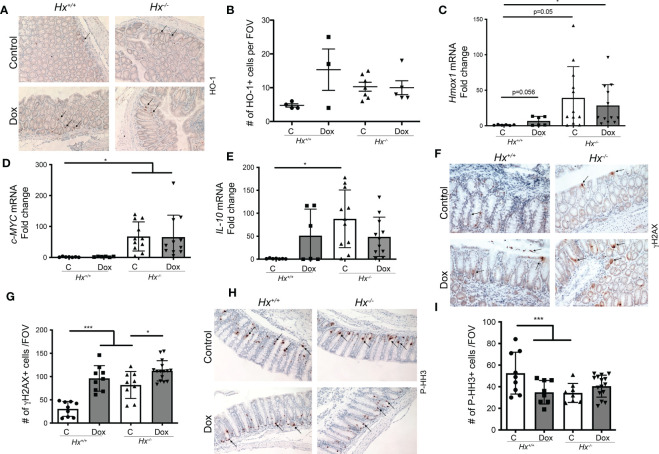
Deficiency in Hx results in altered DNA damage and proliferation in response to Dox treatment in the colon. **(A, B)** Immunohistochemical staining of HO-1 of the colonic tissues from *Hx^-/-^* and WT (*Hx^+/+^*) mice treated with doxorubicin (Dox, 8 mg/kg, *i.v*.) and harvested at day 2. Representative pictures are shown in A and quantification is shown in **(B)**. **(C–E)** Differential gene expression pattern of *Hmox1* **(C)**, *C-MYC* **(D)**, and *IL-10* **(E)** in the colon tissues upon Dox treatment was compared between the WT and *Hx^-/-^* mice by RT-PCR analysis. β-Actin was used as the housekeeping gene. One-way ANOVA or/and t-tests were used to determine statistical significance. Mean values +/- SD are shown. n=6-14 mice per group. *p<0.05. **(F–I)** Immunohistochemical staining of γH2AX (a marker of DNA damage) **(F)** or P-HH3 (a marker of proliferation) **(H)** and of the colonic tissues from *Hx^-/-^* and WT (*Hx^+/+^*) mice treated with Dox and harvested at day 2. 200x magnification. Quantification is shown in G, **(I)** n=7 (*Hx^+/+^*, Control), n=6 (*Hx^+/+^*, Dox), n=13 (*Hx^-/-^*, Control) and n=14 (*Hx^-/-^*, Dox), each evaluated in triplicates. Mean values +/- SD are shown. ***p<0.001, *p<0.05.

To investigate the colonic injury in the absence of Hx in response to chemotherapy, we used doxorubicin treatment (8 mg/kg, 1x, *i.v*.) in *Hx^-/-^* mice (**Figure 5**). We observed increased levels of HO-1+ cells infiltrating into the colon of wild type (WT) mice treated with 8 mg/kg doxorubicin (1x, *i.v*.) (n=3-4 mice/group) and harvested tissues at 48 hours after treatment (**Figure 5A**). The numbers of HO-1+ cells infiltrating colon (**Figures 5A, B**), as well as *Hmox1* mRNA levels in response to doxorubicin treatment, were similar to those seen basally in *Hx^-/-^* mice (**Figure 5C**). Doxorubicin treatment did not increase HO-1 levels in *Hx^-/-^* mice beyond the elevated levels in the untreated *Hx^-/-^* colons. *c-MYC* mRNA levels remained unchanged in response to doxorubicin in WT mice (**Figure 5D**). However, there was a substantial increase in the expression of *c-MYC* in *Hx^-/-^* mice, basally and in response to doxorubicin treatment (**Figure 5D**). We showed elevated *IL-10 mRNA* levels in wild type mice treated with doxorubicin and significantly increased *IL-10* expression in *Hx^-/-^* mice (**Figure 5E**). In this model, we also found significantly higher number of γH2AX+ cells in the colon of doxorubicin treated WT mice (**Figures 5F, G**). Further, an increased number of γH2AX+ cells in *Hx^-/-^* mice with or without doxorubicin treatment was observed (**Figures 5F, G**). Doxorubicin did not lead to increased DNA damage in *Hx^-/-^* model beyond what was seen in the WT mice (**Figures 5F, G**). These data were further supported by slightly decreased levels of P-HH3, a proliferation marker, in the colons of WT mice treated with doxorubicin (**Figures 5H, I**). Lack of Hx resulted in lower proliferation in the colonic crypts, but there was no significant difference between WT and *Hx^-/-^* mice treated with doxorubicin (**Figures 5H, I**). The data described above demonstrate the presence of HO-1+ cells infiltrating the colon in the GIS model, whose number/level is induced by doxorubicin. The presence of these cells also suggests that colonic homeostasis in *Hx^-/-^* mice is altered after treatment with doxorubicin.

### 2.5 Abdominal radiation induces hemolysis and DNA damage in *Hx^-/-^* mice

**Figure 6:**
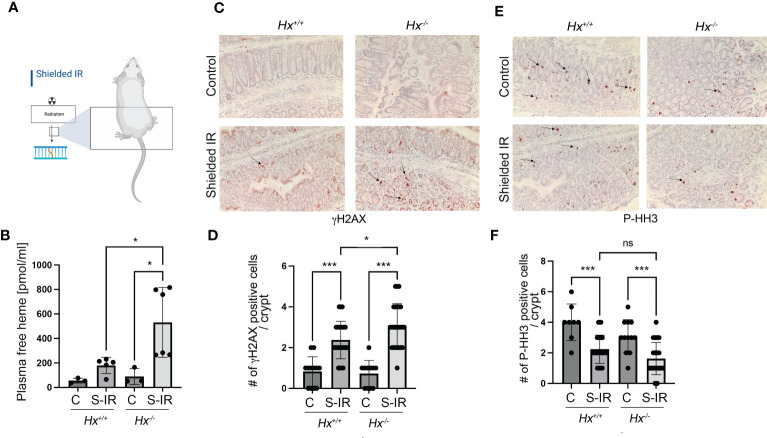
Abdominal radiation induces hemolysis and increased DNA damage in Hx^-/-^ mice. **(A)** A scheme showing the abdominal radiation (shielded IR) procedure in mice. The upper body is shielded, and only the lower part of the body is irradiated. **(B)** Plasma levels of free heme were measured in WT and *Hx^-/-^* mice treated with abdominal irradiation (one dose of 12 Gy) and harvested at 48 hours. n=3 control mice per group, n=6 irradiated mice per group. Mean values +/- SD are shown. *p<0.05. **(C–F)** Immunohistochemical staining of γH2AX (a marker of DNA damage) **(C)** and P-HH3 (a marker of proliferation) **(E)** of the colonic tissues from *Hx^-/-^* and WT mice treated with radiation as above and harvested at day 2. 200x magnification. Quantification of the number of cells positive for γH2AX **(D)** or P-HH3 **(F)** or in the colons of mice treated as described in **(B)** Mean values +/- SD are shown. N=3 control mice per group, n=6 irradiated mice per group. 2-3 sections were analyzed. *p<0.05; ***p<0.001. ns, not significant.

We have previously shown that radiation associated with bone marrow transplant in *Hmox1* deficient mice is associated with persistent DNA damage in the colon (9). To assess the role of heme:Hx in the model of abdominal irradiation (IR), we used local 12 Gy IR (shielded IR) to induce DNA damage in the colon by shielding the upper body of WT and *Hx^-/-^* mice (**Figure 6A**). We showed a slight increase in hemolysis in WT mice treated with abdominal IR (**Figure 6B**), which was significantly elevated in *Hx^-/-^* mice (**Figure 6B**). This increase in free heme in the plasma of irradiated *Hx^-/-^* mice resulted in significantly higher DNA damage and slightly lower proliferation compared to WT mice treated with the same regimen (**Figures 6C–F**). This data suggests a critical role of hemolysis-regulated DNA damage in the colon in response to abdominal radiation.

### 2.6 Heme induces DNA damage and abnormal epithelial cell proliferation

**Figure 7:**
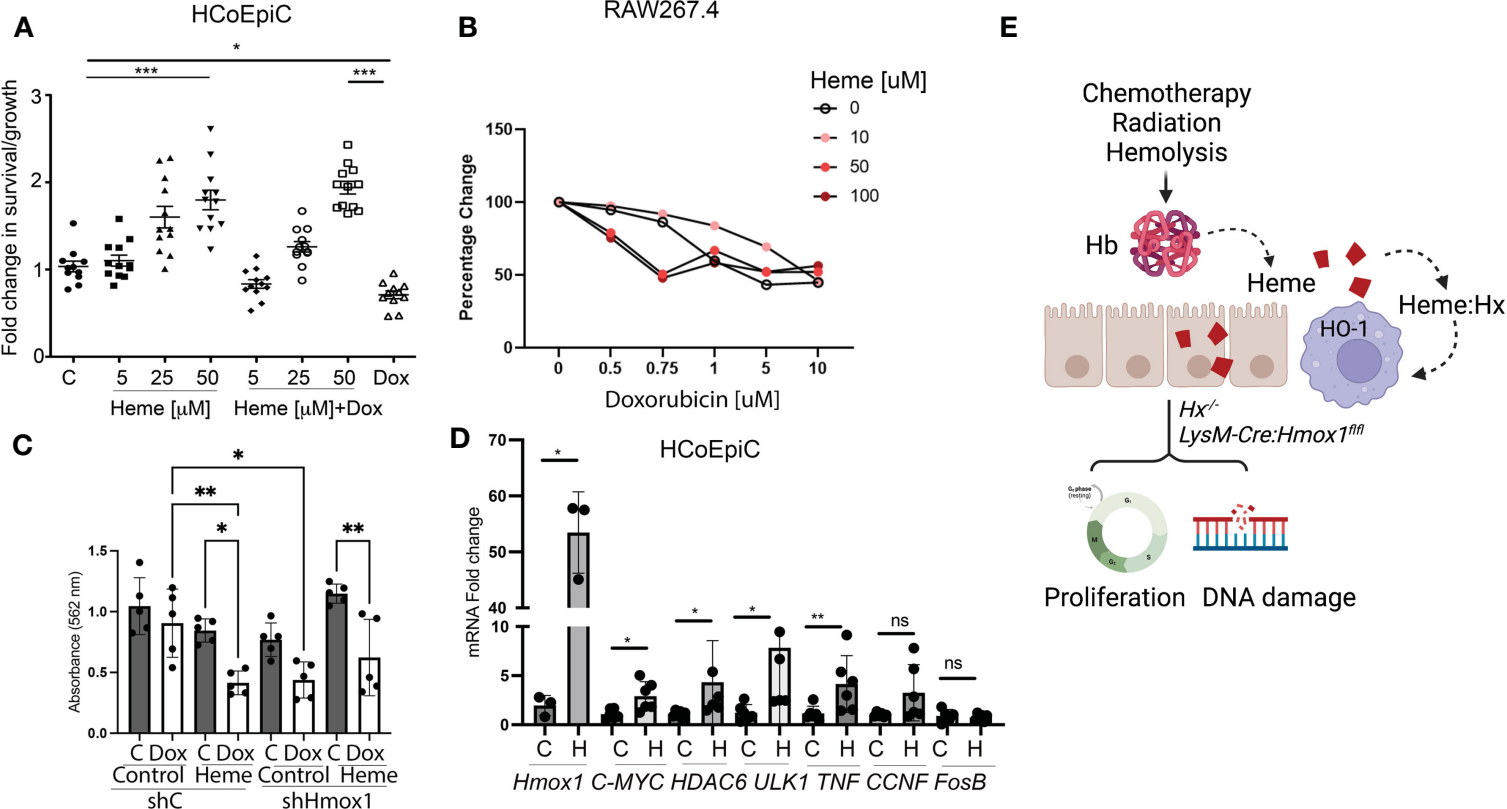
Heme prevents doxorubicin-induced cell death in human colonic epithelial cells but not in Mø. **(A)** HCoEpiC cells were co-treated with Dox (1 μM) and heme (5-50 μM) and assayed for viability after 24 hours of treatment by crystal violet staining. The graph illustrates the percentage change in viability as compared to untreated controls. *p<0.05, ***p<0.001. **(B)** RAW267.4 Mø were co-treated with Dox (0.5-10 μM) and heme (0-100 μM) and assayed for viability after 24 hours of treatment by crystal violet staining. Mean values +/- SD from n=5 replicates are shown. n=12. Data from 2 independent experiments are shown. **(C)** RAW267.4 Mø stably transfected with shRNA against *Hmox1* or scrambled shRNA were co-treated with Dox (0.5-10 μM) and heme (0-100 μM) assayed for viability after 24 hours of treatment by crystal violet staining. Mean values +/- SD from n=5 replicates are shown. Data represenatative for 2 independent experiments are shown. **(D)** Differential gene expression pattern of heme-modulated genes in HCoEpiC cells treated with heme and analyzed by RT-PCR. β-Actin was used as the housekeeping gene. One-way ANOVA or/and t-tests were used to determine statistical significance. Mean values +/- SD; n=6. Data representative for 2 independent experiments are shown. *p<0.05, **p<0.01. **(E)** Scheme depicting the role of free heme released in response to chemotherapy, radiation, or hemolysis in the gut. Lack of HO-1 in macrophage or Hx may result in heme-induced DNA damage and altered proliferation.

Previous reports indicated that heme induces hyperproliferation of epithelial cells in the colon (26). Using human colonic epithelial cells (HCoEpiC), we observed an increase in cell proliferation and survival in response to heme (**Figure 7A**). The survival of heme-treated cells was unchanged upon co-treatment with 1 μM doxorubicin, which decreased the total number of viable HCoEpiC cells as expected (**Figure 7A**). In contrast to colonic cells, heme treatment of Mø (RAW267.4 cells) promoted doxorubicin-induced cell death (**Figures 7B, C**). Further, knockdown of *Hmox1* in the RAW 264.7 macrophage cell line using stable shRNA against *Hmox1* (sh*Hmox1*) resulted in decreased survival in response to doxorubicin in the presence or absence of heme (**Figure 7C**). Heme treatment alone at 1-50 μM doses did not induce cell death as we previously reported (27). We also performed real-time PCR in HCoEpiC cells treated with heme at 24 h to validate changes in G4-regulated genes including *c-MYC, CCNF, HDAC6, ULK1, FosB* as well as a pro-inflammatory cytokine, TNF (**Figure 7D**). *Hmox1* mRNA levels after treatment with heme were increased (**Figure 7D**). We found that heme:G4 complexes-regulated genes such as *c-MYC* and *HDAC6* (12) as well as TNF were increased upon stimulation with heme. In contrast, no differences were found in *FosB* or *CCNF* gene expression levels (**Figure 7D**). These data indicate a strong impact of free heme on normal colonic cell gene expression profile and their proliferation and survival basally and in response to doxorubicin treatment.

## 3 Materials and methods

### 3.1 Patient materials

All studies were approved by the IRB committee at the BIDMC. The archival pathology specimens from the Department of Pathology at BIDMC were identified by a search of Pathology records for the period 2018-2021. H&E stained sections of the samples were reviewed by one of the authors (J.G.) and the pathologic diagnoses were confirmed. The medical records were examined to determine the following patient characteristics: age and stage at diagnosis, adjuvant treatment (chemotherapy and/or radiation), and disease status at the time of last-known follow-up. Colorectal biopsies or resections were collected from patients after radiotherapy for primary malignancy or untreated (including rectal, prostate, colon, lung, and esophageal cancer patients) (n=11 control, n=12 irradiated) and from patients diagnosed with intestinal ischemia (n=11) following the IRB protocol. The control group included patients without gastrointestinal symptoms and no history of chemoradiotherapy or intestinal ischemia, who had histologically normal mucosa in biopsies. All samples were fixed in neutral buffered formalin and embedded in paraffin.

### 3.2 Immunohistochemistry

Pathology specimens were obtained as paraffin blocks and sectioned at 5 μm. Tissue samples were formalin-fixed followed by paraffin embedding, and immunostaining of 5 μm sections was performed as previously described. The following antibodies were used for human material and mouse tissue staining: γH2AX (Cell Signaling, MA), P-Histone H3 (Cell Signaling, MA), and HO-1 (Enzo Life Sciences; Abcam). Tissues were de-paraffinized and processed for antigen retrieval using high-pressure cooking in citrate buffer. Sections were then blocked for 30 min in 7% horse serum (Vector Laboratories, Burlingame, CA, USA). Primary antibody was then applied to the sections overnight at 4°C. The following day, sections were incubated with biotin-labeled (Vector Laboratories, Burlingame, CA, USA) or fluorescently-labeled (Alexa Fluor488 or Alexa Fluor594; Thermo Fisher) secondary antibody for 1 h at room temperature, followed by VECTASTAIN Elite ABC kit and detection with ImmPACT DAB (Vector Laboratories, Burlingame, CA, USA). All images were captured using a Nikon Eclipse E600 microscope (Nikon Instruments, Melville, NY, USA) or Zeiss Fluorescence Microscope. Protein staining was evaluated by both stain intensity and proportion of positive-staining cells. The intensity scale is as follows: 0 (no pigmentation), 1 (light yellow), 2 (buff), and 3 (brown). The percentage of positive cells was assessed by high power field: 0 (<5% chromatic cells), 1 (5–25% chromatic cells), 2 (26–50% chromatic cells), 3 (51–75% chromatic cells), and 4 (>75% chromatic cells).

### 3.3 Animal models

All experimental procedures were performed in accordance with relevant guidelines and regulations and were approved by the Institutional Animal Committee (IACUC) at BIDMC. Male and female C57BL/6 wild-type (WT), as well as *LysM-Cre : Hmox1^flfl^* and *Hmox1^flfl^* mice were maintained in our colony as previously described (28). *Hx^-/-^* mice were a kind gift from Dr. Tolosano (29). For PHZ treatment, the mice were treated with 100-150 mg/kg, intraperitoneal injection (i.p.), at 8-12 weeks of age. In a separate group of mice, gamma irradiation was used at a dose of 12 Gy in the supine position. The upper body was shielded using lead shielding plates (Precision X-ray) covering the head, torso, upper extremities, to the low xiphoid. Regions below the xiphoid were irradiated. All the mice were observed daily for any signs of radiation sickness, morbidity, and mortality. Their body weight was recorded daily. Recombinant Hx was obtained from Athens Research and was used at 2.5 mg/kg, *i.v*. in mice. Animals in the doxorubicin treatment group were treated with a single intravenous (i.v.) dose of 8 mg/kg doxorubicin. Tissues and blood were harvested from the control and treated mice after 12, 24, and 48 h for further analysis. Complete blood count (CBC) was measured using HemaVet 950 analyzer (Drew Scientific, Inc. CT).

### 3.4 RNA isolation and real-time PCR

Total RNA was isolated from mouse colons or epithelial cells using RNeasy Plus Mini Kits (QIAGEN, Valencia, CA, USA), and cDNA was synthesized using HiFiScript cDNA Synthesis Kit (CWBIO) based on manufacturer instructions. To quantify the gene expression levels, synthesized cDNA and PowerUpTM SYBR Green Master Mix (Applied Biosystems) were used to amplify the target genes. Amplifications were performed on 1µg cDNA using the following primers **Table 1**:

**Table 1:** Oligonucleotide sequences used for quantitative real-time PCR.

Genes	Forward Sequence	Reverse Sequence
β-Actin	CCACAGGATTCCATACCCAAGA	TAGACTTCGAGCGACCACATGG
Hmox-1	CAGGATTTGTCAGAGGCCCTGAAGG	TGTGGTACAGGGAGGCCATCACC
C-MYC	GCCCAGTGAGGATATCTGGA	ATCGCAGATCAAGCTCTGGT
HDAC6	TCA GGT CTA CTG TGG TCG TT	TCT TCA CAT CTA GGA GAG CC
ULK-1	CGT CCT CCA AGA CGC TGT AT	CCT GTT GCT TTC CTC CAA AG
TNF	CTGAACTTCGGGGTGATCG	GCT TGG TGG TTT GCT ACG AC
CCNF	AGGACAAGCGCTATGGAGAA	TCTGTCTTCCTGGAGGCTGT
FosB	GCAGGAAGACTGCACAGAAA	AGGAGTCCACCGAAGACAGA
IL-10	TCTCCGAGATGCCTTCAGCAGA	TCAGACAAGGCTTGGCAACCCA

The following program was applied: 95°C for 10 min, 95°C for 15 s, 58°C for 55 s, 72°C for 55 s, 95°C for 15 s, 60°C for 1 min, and 95°C for 15 s (steps #2 to #4 repeated for 40 cycles). StepOne software version 2.3 (Applied Biosystems, MA, USA) was used to calculate relative changes in mRNA levels that were normalized to the β-actin levels.

### 3.5 Cell culture and treatments

The mouse macrophage cell line, RAW 264.7, was purchased from ATCC and maintained in RPMI media (Life Technologies) supplemented with 10% fetal bovine serum (FBS). HCoEpiC (ScienCell Labs) were purchased and maintained in CoEpiCM full medium as specified by the manufacturer’s protocol (ScienCell Labs). Cells were incubated at 37°C with 5% CO_2_.

HCoEpiC cells at passages 3-5 and RAW cells were seeded on 6-well plates at 200 000 cells/well density. Cells were incubated at 37°C with 5% CO_2_ overnight to let them adhere. Cells were treated with 5-50 μM heme with or without doxorubicin for 24 hours. Hemin (referred to as heme, Sigma-Aldrich, St. Louis, MO, USA) was prepared by dissolving the powder in 0.1N NaOH and then titrated with 0.1N HCl to biological pH 7.4, followed by adjustment to 10 mM concentration with 0.9% saline. Heme stock was then aliquoted and frozen at −80°C until use; each aliquot was thawed only once. Heme-utilizing experiments were carried out in the dark at various concentrations of 1–50 µM. Doxorubicin hydrochloride (Sigma-Aldrich, St. Louis, MO, USA) stock (2 mg/ml) was stored in the dark at 4°C until use for cell culture treatment 0.5-10 μM.

### 3.6 Crystal violet staining

HCoEpiC or RAW264.7 cells were seeded on a 96-well plate at a density of 20 000 cells/well. Cells were treated with the following reagents: 5-50 μM heme, 0.5-10 μM doxorubicin, and combined treatment with 5-50 μM heme and/or 1 μM doxorubicin for 24 hours. For the control group (six replicates) only, CoEpiCM complete medium was added. After 24 hours, cells were rinsed with 1xPBS (GIBCO, Life Technologies) and stained with crystal violet (Sigma Aldrich) for 20 minutes on a shaker. After staining, plates were washed in water to remove excess staining. Crystal violet-stained cells were dissolved in 10% acetic acid and absorbance was measured at 560 nm using an ELISA plate reader.

### 3.7 Geo profiles

Genomic profiles of the mouse intestine were obtained from Geo Profiles (24).

### 3.8 Assessing hemolysis and heme levels

Plasma samples were obtained by centrifuging blood (on EDTA) at 1600 x g for 10 min at 4°C. The plasma was then additionally spun at 16,000 x g for 10 min. The levels of heme/hemoglobin were measured as absorbance at 420 nm. The levels of free heme were measured by Heme Colorimetric Kit (BioVision) as previously described (30).

### 3.9 Statistical analysis

All data are presented as mean ± standard deviation unless otherwise indicated. Statistical analysis was performed using Student’s t-test or one-way analysis of variance (ANOVA) followed by the *post hoc* Tukey test using Prism 9.0 (GraphPad Software, San Diego, CA, USA). Differences between groups were rated significant at values of p < 0.05.

## 4 Discussion

In this study, we evaluated the role of free heme in modulating a common adverse effect of genotoxic anti-cancer therapy. Chemotherapy interferes with DNA synthesis and replication, leading to cell death of normal cells (31) and resulting in side effects such as colon inflammation, cardiotoxicity, and myelosuppression (10). Similarly, radiation-induced GIS is an inflammatory disease of the colon in response to anti-cancer treatment characterized by the activation of immune cells, production of pro-inflammatory cytokines, and oxidative stress (32, 33). Both treatment modalities result in the release of DAMPs including free heme (34). Therefore, we hypothesized that the elimination of free heme via HO-1 activity and Hx sequestration may be potential therapeutic approaches to ameliorate the side effects of free heme released in the context of anti-cancer therapy (**Figure 7E**).

We found that active rectal bleeding (and thus higher local heme levels) is associated with higher HO-1+ Mø in the colons of patients with injuries induced by ischemia, radiation, or chemoradiation. HO-1 is a well-established cytoprotective molecule that has antioxidant and anti-inflammatory properties (22, 27, 35–38). HO-1+ Mø exhibits M2-like anti-inflammatory wound-healing phenotype, which may modulate anti-cancer therapy efficacy (22, 39). Studies have suggested that induction of HO-1 by various methods, such as pharmacologic agents, or by administration of recombinant Hx, can enhance the efficacy of doxorubicin and reduce its toxicity (26, 40, 41). For example, increasing HO-1 expression has been shown to reduce doxorubicin-induced cardiotoxicity in animal models (42, 43). Similarly, administration of Hx to wild-type (WT) mice treated with doxorubicin and observed improved cardiac function (26). We have previously reported that HO-1 overexpression facilitates chemotherapy-induced cancer cell death while protecting normal cells (10, 12). We did not see any changes in γH2AX staining between patients after CRT with or without rectal bleeding. However, we detected a positive correlation between γH2AX and HO-1+ Mø staining in the colons of these patients (p=0.013, r^2^ = 0.443). This was independent of epithelial HO-1 expression. The small number of subjects and the lack of detection of free heme in human samples may limit our ability to detect the impact of bleeding/HO-1+ Mø staining on DNA responses and colonic injury in our studies. Further work will address these questions in larger cohorts.

The work in our current study demonstrated that myeloid-specific *Hmox1* knockout mice exhibited increased DNA damage and proliferation of epithelial cells in the colons upon induction of hemolysis (**Figure 7E**). This observation is direct evidence that elevated heme levels due to limited HO-1 activity lead to colonic injury. Recruitment of monocytes and Mø to the injury site may be considered detrimental to colon recovery. Activated Mø (44), produce pro-inflammatory cytokines and reactive oxygen species that further propagate inflammation and tissue damage (44). However, heme-induced HO-1+ Mø possess anti-inflammatory properties and promote tissue repair (45, 46). Recent studies have suggested that induction of *Hmox1* expression in Mø in response to heme may serve a protective function by mitigating colonic inflammation and damage in animal models of colitis (23). Some others have suggested enteral hemin therapy as a potential treatment for colitis (23). We have previously reported that HO-1 is highly expressed and contributes to the M2 phenotype. Deletion of HO-1 promotes an M1-like phenotype (27, 47). In the acute colitis model, HO-1 is mainly localized in F4/80-immunopositive and CD11b-immunopositive Mø (48). In another study using the DSS model, the colonic Mø expressed HO-1 that was stained positive for CD68 (49). HO-1 has been shown to localized in F4/80+ and CD11+ Mø (48) with an M2-like phenotype. We showed that HO-1 co-stained with CD68+ Mø in human ischemic colon samples. While it is difficult to definitively confirm whether the observed Mø are residential or infiltrated at a single time point in pre-fixed tissues, we speculate that there will be a significant infiltration of HO-1+ Mø that have migrated to the colonic wall. Based on our observation of a low baseline level of HO-1+ Mø, we anticipate that there could be a significant influx of these Mø in response to ischemia. Heme has been shown to promote the infiltration of HO-1+ Mø in other models, which could suggest that heme may have a similar effect in the context of GIS (50).

Our data suggest that in the context of hemolysis and associated colonic injury, the lack of HO-1 results in a predominantly toxic effect of heme. Interestingly, hemolysis in mice bearing macrophage-specific *Hmox1* deletion enhanced epithelial cell proliferation. Similarly, heme promoted the growth and survival of epithelial cells *in vitro*. This effect is related to increased HO-1 levels and other pro-inflammatory and pro-proliferative genes. The knockdown of *Hmox1* in Mø decreased cell survival in response to doxorubicin. Ferroptosis has been consistently reported as a mechanism of doxorubicin-induced cardiac toxicity in patients (51). *Hmox1* induction was shown to contribute to doxorubicin-induced efficacy and toxicity due to changes in free iron, lipid peroxidation, and oxidative stress (52). It has been reported that *Hmox1* overexpression increased the expression of ferritin and transferrin receptors, resulting in the alteration of the intracellular iron distribution and providing a protective effect against iron cytotoxicity from heme degradation (53). Furthermore, Liu et al. show that (26) Hx may also mitigate doxorubicin-induced ferroptosis *in vivo*. Hx-expressing Mø contribute to the reduction of anthracycline-induced inflammation (26). As multiple mechanisms likely contribute to increased survival of HO-1+ Mø in response to doxorubicin, further investigation will be necessary into the mechanisms of doxorubicin-associated colonic injury. We have recently shown that the direct interaction of heme with DNA G-quadruplexes (G4) leads to altered gene expression in cancer cells that regulate transcription, recombination, and replication (11). Here, we provide new data supporting the same mechanism in colonic epithelial cells. We found elevated levels of G4:heme complexes-regulated genes such as *c-MYC*, *CCNF*, *HDAC6*, and *TNF* in HCoEpiC cells. These genes are strongly associated with proliferation/survival and inflammatory stress.

Our data suggest that Hx plays a central role in colonic homeostasis. It has been demonstrated that Hx may revert to HO-1-induced pro-inflammatory activation of Mø (54). However, consumption of Hx occurs in chemotherapy-associated colitis due to increased intravascular hemolysis (55). Intravascular hemolysis appears to be an important contributor to intestinal mucosal damage (56). After PHZ treatment, we found heightened levels of hemolysis, colonic damage, and inflammation in mice lacking the heme scavenger protein, hemopexin. *Hx^-/-^* mice had increased colonic expression of key anti-inflammatory genes (*Hmox1, Il-10*), but poor proliferation and increased DNA damage indicated that Hx may play a role in maintaining gut homeostasis and the regenerative capacity of colonic epithelial cells. We found elevated levels of *c-MYC* in *Hx^-/-^* mice treated or untreated with doxorubicin, which might be an interesting oncogenic signal in the colons of *Hx^-/-^*. The significance of this increase needs to be studied in future work. IL-10 was promptly induced in response to doxorubicin but not significantly. Interestingly, the increase in IL-10 in *Hx^-/-^* basally was significant and most likely related to elevated levels of HO-1 in *Hx^-/-^* (due to free heme). The HO-1/IL-10 axis is well-described in the field (57, 58). It has been shown that Hx levels increased throughout the progression of colitis (59). Our data support this observation. Specifically, we found strong stromal staining of Hx in response to radiation treatment. Our study is the first to show the role of Hx in radiation-induced hemolysis and DNA damage in the colon. Therefore, it is plausible that Hx plays a major role in protecting colonic epithelial cells from heme toxicity.

While heme has been widely used to induce *Hmox1* expression, no data are available on the protective role of HO-1 in the intestinal barrier disruption after doxorubicin treatment, mimicking the physiological process of colitis. We found that doxorubicin increased the expression of *Hmox1* in wild-type mice, as we previously reported in cancer cells (10). One classical feature of tumorigenesis is the metabolic acquisition of a highly glycolytic phenotype. Carbon monoxide (CO), one of the products of HO-1 has been implicated in carcinogenesis and therapeutic resistance in several tumor cell types (10). However, the functional contributions of CO and HO-1 to these processes are poorly defined. In human prostate cancers, we found that HO-1 was nuclear localized in malignant cells, with low enzymatic activity in moderately differentiated tumors correlating with worse clinical outcomes. Exposure to CO or overexpression of enzymatically active HO-1-sensitized prostate cancer cells but not normal cells to chemotherapy, with growth arrest and apoptosis *induced in vivo*. CO targeted mitochondria activity in cancer cells as evidenced by higher oxygen consumption, free radical generation, and mitochondrial collapse (10). In the present work, we did not see a major effect of Hx deletion on doxorubicin-induced DNA damage. While deletion of Hx does not significantly affect DNA damage caused by doxorubicin *in vivo*, the results highlight the important role of Hx in protecting the colon from heme injury. Our previous work indicates that a lack of Hx in the stroma facilitates tumor growth (12). We will address in future studies whether lack of Hx impacts anti-cancer therapy efficacy. Similarly, the potential beneficial effects of HO-1 induction may vary depending on the type of cancer and the specific treatment regimen. The balance between efficacy and toxicity must be carefully considered when determining the optimal use of HO-1 or Hx-modulating agents in radiochemotherapy.

In conclusion, the fluctuation in the heme levels and expression of *Hmox1* may help to understand the incidence of gastrointestinal injury (GIS, gastrointestinal syndrome) related to CRT. In this study, we confirmed previously reported results (15) that heme-induced DNA damage, proliferation, and inflammation in human colonic epithelial cells. We show that PHZ- or abdominal radiation-induced hemolysis can cause damage in the colon. This colonic damage is partially regulated by the activity of HO-1-positive Mø and Hx. Our results suggest that the protective role of Hx and HO-1 may be important in hemolysis-induced colonic injury or patients with active rectal bleeding. The role of HO-1 and Hx in mediating the side effects of doxorubicin therapy is still the subject of ongoing research and further studies are needed to fully understand its potential as a therapeutic target.

Collectively, we demonstrated that heme accumulation in the context of anti-cancer therapy can contribute to colonic injury. These mechanisms suggest that both HO-1 induction and Hx substitution may have therapeutic potential to ameliorate the side effects of anti-cancer therapy.

## Data availability statement

The raw data supporting the conclusions of this article will be made available by the authors, without undue reservation.

## Ethics statement

The studies involving human participants were reviewed and approved by BIDMC IRB. Written informed consent for participation was not required for this study in accordance with the national legislation and the institutional requirements. The animal study was reviewed and approved by BIDMC IACUC.

## Author contributions

BW, PS, MJ, and SA designed the study. BW conceived the original idea. MJ, LJ, and SA acquired an original set of *in vitro* and *in vivo* data. BW, and JG performed the analysis of patient samples and clinical correlation. PS, MJ, LJ, MO’C, and SA performed *in vivo* experiments. EC and SA performed immunohistochemistry (IHC) and immunofluorescence (IF) staining. PS, SA, and BW analyzed the data. MJ, SA and PS performed colony assays with genetic quality control (GQC) and part of the western blots. PS, and BW wrote the paper with input from SR. BW supervised the work. All authors contributed to the article and approved the submitted version.
