# Supplementary material for: Free heme exacerbates colonic injury induced by anti-cancer therapy
Source: Front Immunol. 2023 Jun 5;14:1184105. doi: 10.3389/fimmu.2023.1184105 (PMC10277564; doi:10.3389/fimmu.2023.1184105)
Supplement: Supplementary file 1 [file DataSheet_1.pdf]

Supplementary Material

Free heme exacerbates colonic injury induced by anti-cancer therapy

Philippa Seika[1,#], Monika Janikova[1,2,#], Sahana Asokan[1,3,4,#], Lubica Janovicova[1,2], Eva Csizmadia[1], Mckenzie O'Connell[1], Simon C. Robson[5], Jonathan Glickman[6, 7] & Barbara Wegiel[*,1]

*** Correspondence:** Barbara Wegiel: bwegiel@bidmc.harvard.edu

1 Supplementary Data

2 Supplementary Figures and Tables

2.1 Supplementary Figures

Supplementary Figures:

Supplementary Figure 1

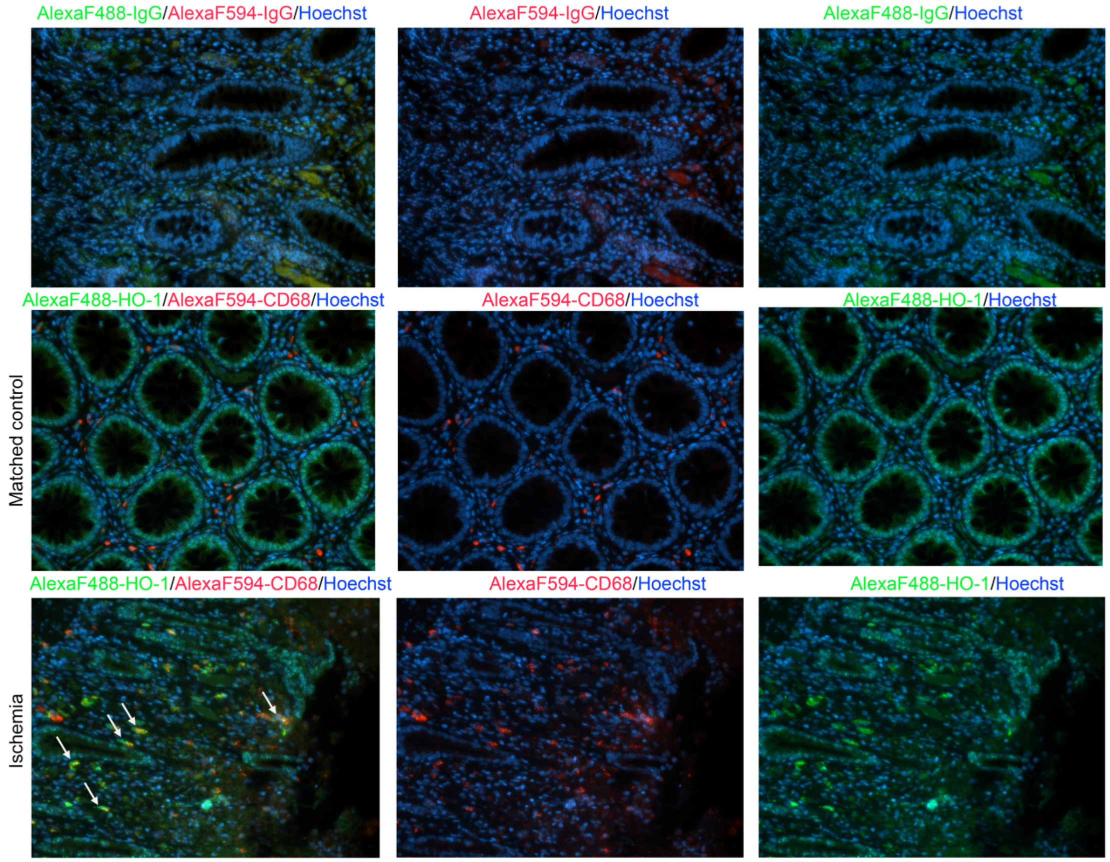

Supplementary Figure 1. Immunofluorescence staining of HO-1 and CD68 in human intestinal biopsies from patients suffering from intestinal ischemia as in Fig. 1A. Staining is representative for n=4 patients with ischemic colons and matched normal mucosa. Arrows indicate HO-1+CD68+ Mø.

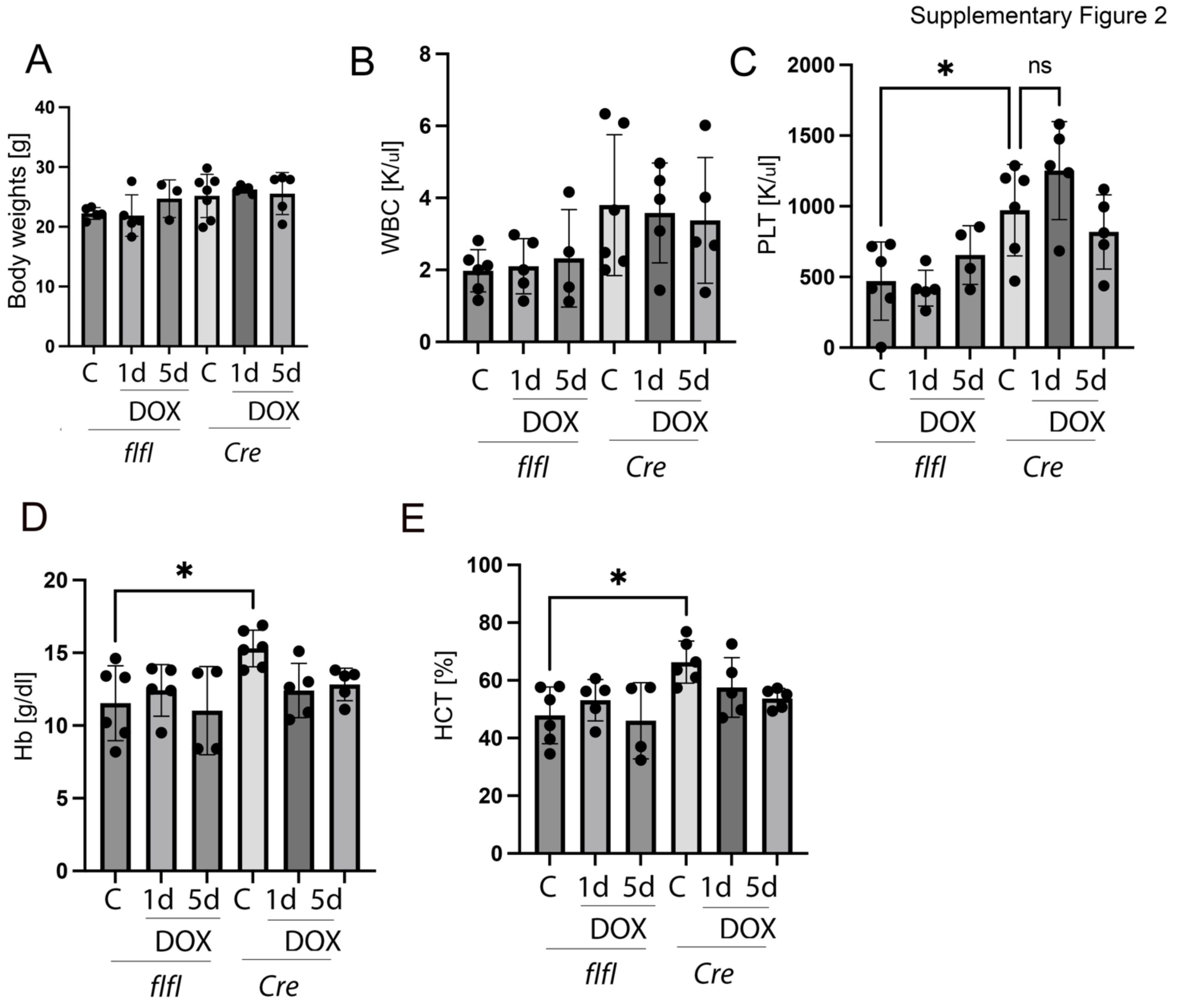

Supplementary Figure 2. Body weights and blood analyses of *LysM-Cre:Hmox1*^{flfl} mice treated with doxorubicin. *LysM-Cre:Hmox1*^{flfl} and *Hmox1*^{flfl} mice were treated with doxorubicin (8 mg/kg, i.v.) and harvested at 1 or 5 days after treatment. Body weights are shown in **A** and blood count analysis is shown in **B-E**. *p<0.05.

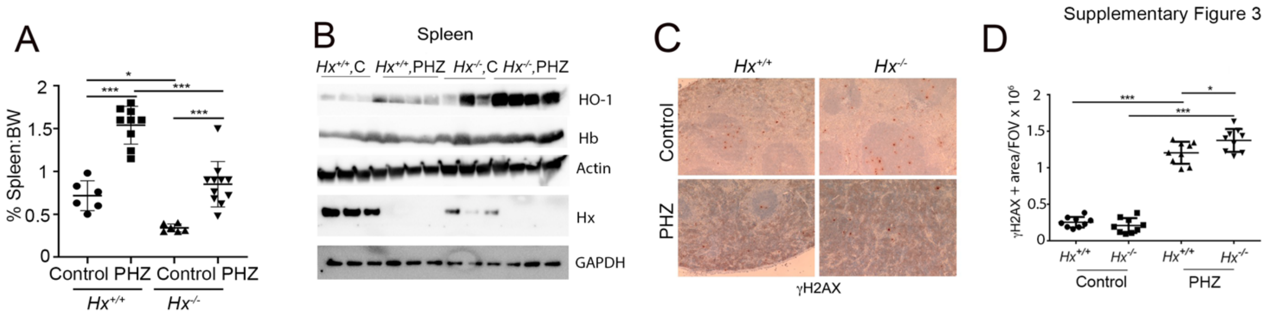

Supplementary Figure 3. Splenic phenotype of Hx KO mice. Spleen: BW (body weight) ratios (A), Western blotting with antibodies against HO-1, Hx and hemoglobin in the spleens (B), and immunohistochemical analysis of γH2AX (a marker of DNA damage) of the splenic tissues (C-D)

were assessed in *Hx*$^{-/-}$ and *Hx*$^{+/+}$ mice were treated with PHZ (100 mg/kg, *i.p.*) for 48 h. ***$p<0.001$, *$p<0.05$.
